# Patient compliance with Twin Block appliance during treatment of Class II malocclusion: a randomized controlled trial on two check-up prescriptions

**DOI:** 10.1093/ejo/cjac046

**Published:** 2022-08-15

**Authors:** Erik Frilund, Mikael Sonesson, Anders Magnusson

**Affiliations:** Department of Orthodontics, The Institute for Postgraduate Dental Education, Jönköping, Sweden; Centre for Oral Health, School of Health and Welfare, Jönköping University, Jönköping, Sweden; Centre for Oral Health, School of Health and Welfare, Jönköping University, Jönköping, Sweden; Department of Orthodontics, Section 4, University of Malmö, Malmö, Sweden; Department of Orthodontics, The Institute for Postgraduate Dental Education, Jönköping, Sweden; Centre for Oral Health, School of Health and Welfare, Jönköping University, Jönköping, Sweden; Department of Biomedical and Clinical Sciences, Linköping University, Linköping, Sweden

## Abstract

**Background:**

Compliance is crucial for the treatment outcome with removable appliances. Previous studies on treatment with the Twin Block appliance have focused on effectiveness in relation to other treatment methods or wear-time. Studies on different check-up intervals to improve compliance seem to be lacking.

**Objectives:**

To compare the impact of two different check-up prescriptions on patient compliance and treatment outcome during treatment with Twin Block.

**Trial Design:**

Two-arm parallel group, single-centre, randomized controlled trial.

**Materials and Methods:**

Seventy-three patients, 38 boys, and 35 girls, mean age 11.2 years, were included and block-randomized into two groups treated with a Twin Block appliance. Group 1 was called for check-up visit every sixth week and group 2 every fourth week. Compliance was evaluated with a TheraMon® microsensor, moulded into the appliance, measuring wear-time. Overjet, overbite, and molar relationships were assessed on study casts before and after treatment. The treatment outcomes were analysed on an intention-to-treat basis.

**Results:**

In group 1, the reduction of overjet was 5.2 mm and the mean wear-time was 6.9 hours. In group 2, the reduction was 4.7 mm and the wear-time was 6.1 hours. Seventy-four per cent of the patients presented an overjet of 4 mm or less. Wear-time did not correlate to age, gender, or severity of malocclusion.

**Harms:**

No harm was observed in any patient. Lateral open bites were registered during treatment but were normalized at the end of the treatment.

**Limitations:**

The trial was a single-centre study and long-term effects were not evaluated.

**Conclusions:**

During treatment with the Twin Block appliance, a 4-week check-up interval did not improve treatment outcome or increase wear-time, compared to a 6-week check-up interval. The mean wear-time was 6.5 hours per day, even if the recommendation was 12 hours.

**Clinical Trial Registration:**

NCT05155774

## Introduction

A Class II malocclusion with excessive overjet and incomplete lip closure is one of the most common malocclusions and affects between 14 and 25 per cent of Scandinavian children (1,2). The malocclusion may increase the risk of trauma to the maxillary incisors and reduce the oral health-related quality of life in children (3–5). Early treatment in the mixed dentition seems to be successful to reduce sagittal and vertical discrepancy and to improve lip closure (6,7). Several types of functional appliances are available. The Twin Block appliance has been used to treat patients with Class II malocclusion, excessive overjet, and incomplete lip closure (4,8–10).

Successful treatment with a removable appliance is dependent on wear-time and patient compliance. If compliance fails, the treatment outcome is jeopardized and this incurs costs for the dentist, patient, and society (11,12). In previous studies on functional appliances, approximately 25 per cent of treatments did not fulfil their objectives (6). To encourage compliance, patients are commonly scheduled for regular visits at the dental clinic. The recommended check-up interval during treatment with a Twin Block appliance is 6–8 weeks (13,14). To increase compliance, a shorter interval between the check-ups has been suggested (15,16). It may be a challenge to estimate patient compliance with a removable appliance and it is common for the patient to overestimate the wear time (17,18). There is conflicting evidence concerning how compliance is affected by age or growth (19,20). To objectively measure patient compliance, the TheraMon® microsensor (Handelsagentur Gschladt, Hargelsberg, Austria) has been introduced (21). The sensor records the patientʹs wear-time by registering the temperature in the oral cavity at regular intervals (22–24).

Randomized controlled trials (RCTs) on optimal check-up intervals to increase compliance with the Twin Block appliance seems, to our knowledge, be missing in the literature. Thus, the present study was an RCT on patient compliance with Twin Block, assessed by a TheraMon® microsensor, and the primary aim was to compare how 6- and 4-week check-up intervals affect wear-time, as well as changes in overjet. The secondary aim was to compare the treatment effects of the two check-up intervals on overbite and molar relationship. The null hypothesis was that there would be no difference in treatment outcome between the two groups.

## Subjects and methods

Eighty-eight patients were assessed for eligibility in the present RCT. The study was designed with two parallel arms comparing 6- and 4-week check-up intervals and treatment outcomes. The trial was carried out at The Institute of Postgraduate Dental Education at the Department of Orthodontics, Jönköping, Sweden, in collaboration with the public dental health service clinics in Jönköping County Council, Sweden. The study was approved by the ethical board at the University of Linköping, Linköping, Sweden, (Dnr. 2017/382-31). During the start of the study, a registration at www.ClinicalTrials.gov (NCT05155774) was performed.

### Patients and eligibility criteria

The patients were recruited and treated from December 2018 to October 2020. The inclusion criteria were healthy patients according to the American Society of Anaesthesiology Classification (ASA I and II), aged 8–14 years at treatment start and with an overjet of 6 mm or more together with an incomplete lip closure. Exclusion criteria were severe chronic conditions such as asthma and allergy, neuropsychiatric disorders, craniofacial syndromes, and previous orthodontic treatment. Recruitment of the patients continued until the number of participants met the estimated sample size.

### Randomization and intention-to-treat

The patients and the legal custodians were given verbal and written information about the study by an orthodontist (AM). The participants were randomly allocated into one of two check-up groups after receiving informed consent from the patients and their custodians. The patients in group 1 were checked every sixth week and the patients in group 2 were checked every fourth week. The randomization was done by a computer-generated block-randomization list and provided by a staff member not involved in the treatments of the patients (25). The list consisted of nine blocks with 10 patients in each block. Data on all patients were evaluated on an intention-to-treat (ITT) basis. Accordingly, the dropouts were included in the final analysis by adopting the minimum values for the primary outcomes, as well as for the secondary variables.

### Blinding

Neither the patient nor the operator was blinded to the group assignment. However, the study casts were anonymized, and the examiner (AM) was blinded for the allocation groups when assessing the study casts.

### Treatment and intervention

After randomization and before treatment start (T0) a clinical examination and a regular orthodontic registration were performed at the department of orthodontics. The registrations included study casts and intra- and extra-oral photographs. Dental panoramic radiographs were taken before and after treatment. The treatment protocol was comprised of the two check-up intervals at the public dental health service clinics. A final registration was performed at the department of orthodontics after the treatment was ended (T1).

**Figure 1.**
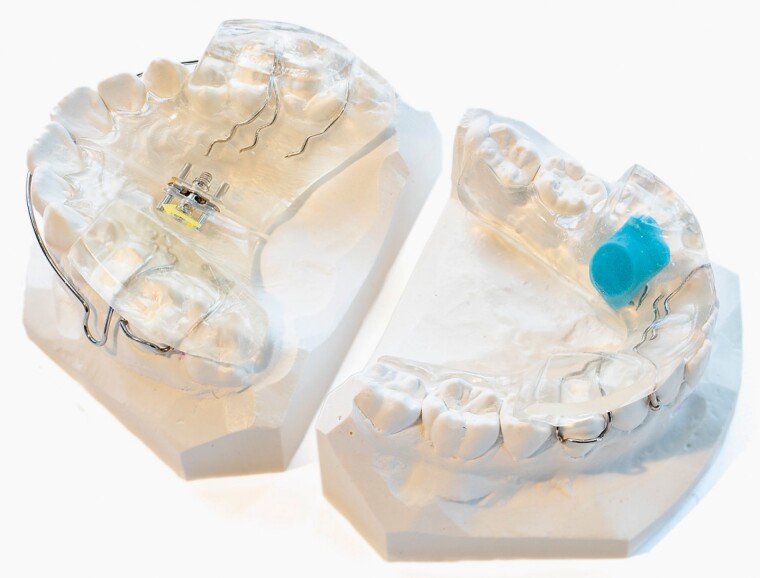
Twin Block appliance with the TheraMon® microsensor in the mandibular lingual section.

The patients were treated with a Twin Block appliance according to Clark *et al.* (2002) (Figure 1) (14). The patients were given verbal and written instructions by an experienced orthodontist (AM) to wear the appliance for at least 12 h a day (13).

The wear-time, overjet, overbite, and molar relation was recorded every 12th week at the department of orthodontics. The wear-time was assessed by scanning a TheraMon® microsensor, which was moulded into the acrylic base of the mandibular lingual section of the Twin Block appliance (21–24). The treatment objectives were an overjet equal to or less than 4.0 mm and Class I molar relationship. The appliances were collected by the orthodontic clinic after treatment and the microsensors were sent for recycling.

### Primary outcome measures

The primary outcomes of wear-time and overjet were compared between the two groups. The wear-time was monitored for up to 12 months of treatment. The wear-time was registered by the TheraMon® microsensor every 20th minute. The sensor was set to measure oral temperatures between 30 and 42°C (24). The data in the sensor were transferred to a computer and converted by software into wear-time (hours per day) (TheraMon® software, version 2.1.0.13) (21,26–29). The participants and their custodian were informed about the monitoring of wear-time.

The overjet was measured on the study casts performed before treatment start (T0) and at the end of treatment (T1) with a digital calliper (Mitutoyo 500-171, Kanagawa, Japan). The measurements were made to the closest 0.1 mm.

### Secondary outcome measures

The overbite was measured in millimetres on the casts before treatment start (T0) and after treatment (T1) with the digital calliper. The measurements were made to the closest 0.1 mm. The molar relationship was examined on the casts.

Successful treatment outcome was a full Angle Class I molar relationship and an overjet of 4 mm or less. Unsuccessful treatment outcome was a persisting Class II molar relationship or a half Class II molar relationship together with/or a remaining overjet greater than 4.0 mm.

Data on trauma to the maxillary incisors before and during treatment were registered from the patient's dental records as well as the total number of check-ups, cancellations, and relocations. In addition, the influence of age on the treatment outcomes was analysed by dichotomizing all included patients into two groups: 8–10 years and 11–14 years.

### Sample size calculation

As no RCT has previously evaluated the impact of the two check-up prescriptions on the reduction in overjet and wear-time, we decided that a measurable difference in overjet of 1 mm (SD 1.6) between the groups was of clinical interest (29). The level of significance was set to *P* < 0.05 and the power to 80 per cent. The sample size calculation showed a need for 40 patients in each group when a 20 per cent drop-out has been considered (30).

### Statistical analysis

The data were tested using independent *t*-test for equality of means, to evaluate any statistically significant difference between the two groups. A chi-squared test and group cross tabulation were used to compare changes in Angle classification between the groups. Group cross tabulation was used to compare changes in horizontal overjet between the two groups. Potential relations between overbite and age, wear-time and age, as well as reduction of overjet and age were conducted using scatterplots. Statistical analysis was performed using SPSS Statistics version 27 (SPSS, Inc., Chicago, Illinois, USA) and the results were deemed to be significant if *P* < 0.05 with a confidence interval of 95 per cent.

### Method error analysis

The intra-class correlation coefficients (ICC) was used to measure the intra-observer reliability of the observer (AM). On 10 randomly selected study casts obtained before (T0) and after treatment (T1) overjet, overbite, and molar relationship were re-measured after 2 weeks. The ICC of the measurements on overjet was 0.77, overbite was 0.87, and molar relationship 0.8, which indicate a good intra-observer reliability.

## Results

**Figure 2.**
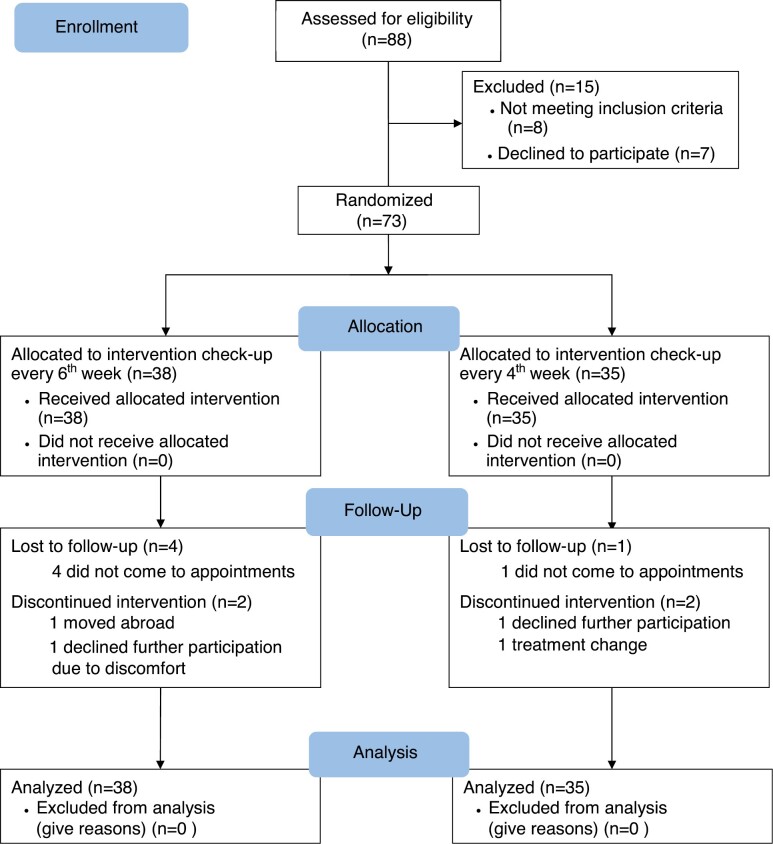
CONSORT flow-chart for RCT. *RCT*, randomized controlled trial.

Eighty-eight patients were primarily assessed for eligibility. Seven patients declined to participate in the study and eight patients were not eligible due to their changed therapy plans. The remaining 73 patients were randomized into the two groups. In group 1, with check-ups every sixth week, 38 patients (22 boys and 16 girls) were included and in group 2, with check-ups every fourth week, 35 patients (16 boys and 19 girls) were included (Figure 2). In group 1, the mean age at treatment start was 10.9 years (SD 1.4) and in group 2, the mean age was 11.6 years (S.D 1.9). No significant age-difference between the groups was seen.

### Primary outcome measures

There was a significant reduction in overjet in both groups during treatment (*P* < 0.05). The mean overjet at T0 was 8.2 mm in group 1 and 8.4 mm in group 2. Mean reduction of overjet was 5.2 mm in group 1 and 4.7 mm in group 2 (Table 1). The mean overjet at T1 was 3.0 mm in group 1 and 3.8 mm in group 2. No significant difference in reduction of overjet was found between the two groups.

**Table 1.** The treatment protocol comprised, according to the randomization, two check-up intervals; (1) every sixth week (group 1) and (2) every fourth week (group 2).

	Group 1 (*n* = 38) 22 M/16 F		Group 2 (*n* = 35) 16 M/19 F		P
	Check-ups every sixth week		Check-ups every fourth week		
	Mean	SD	Mean	SD	
Wear-time (h/day)	6.9	2.8	6.1	2.4	NS
Reduction in overjet (mm)	5.2	2.2	4.7	2.1	NS
Reduction in overbite (mm)	1.2	1.1	0.8	1.3	NS
Total treatment-time (years)	1.1	0.4	1.2	0.3	NS

*NS*, not significant.

Twenty-five patients had an overjet of 9 mm or more at T0. There was no statistical difference in total wear-time compared to the 39 patients with an overjet of 6–9 mm at T0.

**Figure 3.**
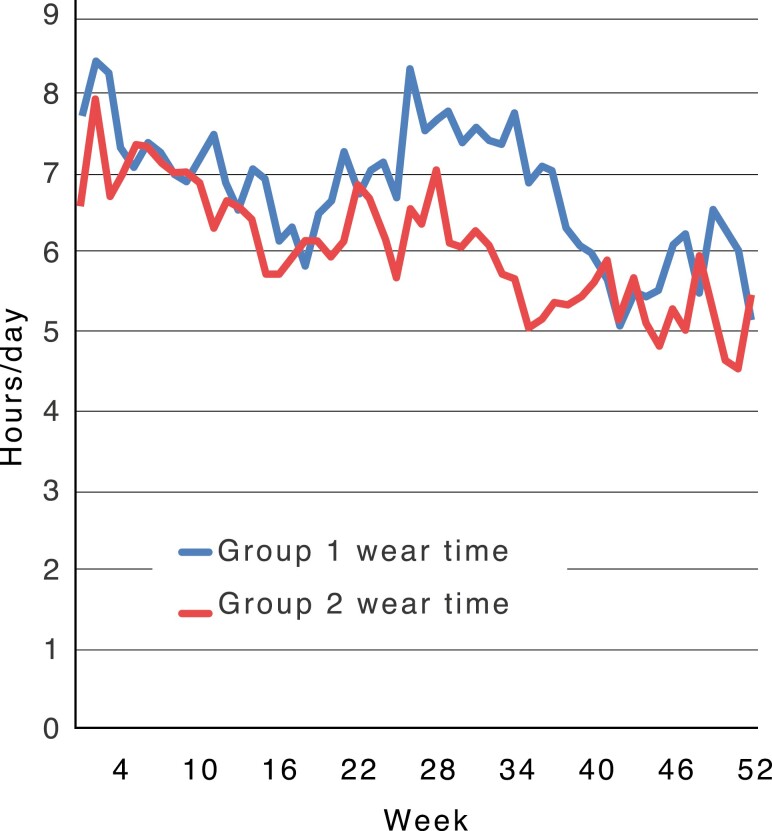
Mean wear-time, first year of treatment in group 1 and group 2.

The mean wear-time in group 1 was 6.9 h per day and in group 2, it was 6.1 h per day. There was no statistically significant difference in wear-time between the two groups. The mean wear-time decreased in both groups during the treatment, except for the period between month 6 and 9, when the mean wear-time in group 1 was like the first 6 months (Figure 3).

### Secondary outcome measures

The reduction of overbite was 1.2 mm in group 1 and 0.8 mm in group 2 (Table 1). All patients had, at treatment start, a Class II molar relationship. The molar relationship after treatment was significantly improved in both groups. An Angle Class I molar relationship was achieved in 26 patients in group 1 and in 19 patients in group 2. Half-cusp molar relation was achieved in 6 patients in group 1 and in 13 patients in group 2. Six patients in group 1 and 3 patients in group 2 remained in Class II molar relationship. The patients that remained in Class II molar relationship did not complete the treatment (Table 2).

**Table 2.** Treatment outcome related to molar relation and overjet.

	Group 1 (*n* = 38) Overjet Post Tx	Group 2 (*n* = 35) Overjet Post Tx	Total *n*
Class I molar relation	Mean 3 mm 26 Cases	Mean 3 mm 19 Cases	45
Half-cusp molar relation	Mean 5 mm 6 Cases	Mean 5 mm 13 Cases	19
Class II molar relation	Mean 8mm 6 Cases	Mean 9 mm 3 Cases	9

Nineteen of the 73 patients (26 per cent) had an experience of traumatic injury to the permanent maxillary incisors before or during treatment (13 boys and 6 girls). Of 39 patients with overjet ≥ 9 mm, 7 children had experienced a traumatic injury. Of 25 patients with an overjet < 9 mm, 12 children had experienced a traumatic injury. No significant difference in prevalence of trauma was seen between patients with an overjet ≥ 9 mm and patients with an overjet < 9 mm. Trauma was significantly more common for boys, where 13 of 31 boys had experienced a traumatic injury compared to 6 of 27 girls.

There was no significant difference related to age and treatment outcomes. The total number of check-up appointments were 308 in group 1 and 384 in group 2. The mean number of check-up appointment per patient in group 1 was 9.6 and 12 in group 2. Of 308 visits in group 1, 16 were cancellations, and 39 were appointment relocations. Of 384 visits in group 2, 24 were cancellations, and 45 were appointment relocations. No statistical differences between the groups concerning cancellations or appointment relocations were seen.

### Successful treatment

**Figure 4.**
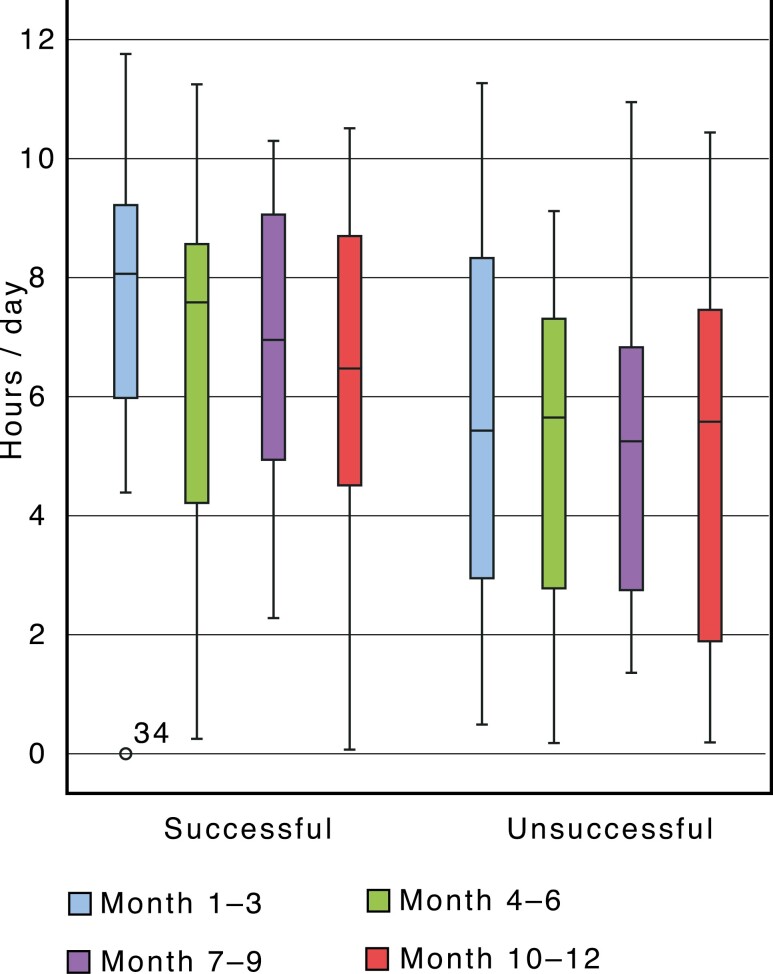
Mean wear-time related to successful treatment in total sample.

In the total sample (group 1 and group 2), the mean wear-time for patients with successful treatment was 6.8 hours per day (range 5.5–9.1) compared to 5.2 hours per day (range 3.1–6.5) for patients with unsuccessful treatment the first year of treatment (Figure 4).

The overjet was reduced to 4.0 mm in 54 of the 73 patients, that is, 74 per cent. The molar relationship was normalized into Class I relationship in 45 of the 73 patients, that is, 62 per cent. Thus, according to the ITT approach, the Twin Block appliance reduced the overjet to less than 4.0 mm and normalized molar relationship in 59 per cent of the patients during the trial. Mean age for patients with successful treatment was 11 years, and 11.5 years for patient with unsuccessful treatment. There were 22 girls and 21 boys in the successfully treated group, and 11 girls and 12 boys in the unsuccessfully treated group. Successful treatment did not correlate to the severity of the initial overjet. Successful treatment was not dependent on gender or age at start of treatment. There was no correlation between the initial overjet and wear-time.

## Discussion

### Main findings

This RCT on treatment with the Twin Block appliance showed no significant difference in treatment outcomes when comparing a check-up interval of 6 weeks (group 1) to a check-up interval of 4 weeks (group 2). Thus, the null hypothesis was confirmed.

During the period of 12 months, the mean wear-time was 6.9 hours per day in group 1 and 6.1 hours in group 2 (Table 1). Compared to the recommended wear-time of at least 12 hours per day, the mean wear-time in both groups was below 55 per cent of the recommended wear-time. Patients seem to wear the appliance mainly during the nights due to speech difficulties and/or inconvenience in leisure-time activities. Similar differences in recommended wear-time and actual wear-time have been reported in earlier studies on treatments with Twin Blocks or other kinds of removable appliances (17,31,32).

There were no significant differences in cancellations or relocations between the two groups. Hence, cancellations and/or relocations should not have affected the control interval between the groups.

The mean wear-time in both groups decreased during the treatment. This is in accordance with earlier studies presenting a negative correlation between wear-time and treatment duration (33–35). Some variations in and between the groups was, however, seen. The patients in group 1 (check-up every sixth week) had an initial decrease in mean wear-time, improved after approximately 6.5 months of treatment, then decreased again. Group 2 (check-up every fourth week) showed a more constant tendency in decline of wear-time from treatment start (Figure 3). However, no significant difference was found between the groups. An explanation for the tendency of more constant decrease in wear-time in group 2 might be that a more frequent control of the appliance tires the patient and reduces their motivation to wear it. However, as mentioned in a previous study, the fluctuating nature of compliance is dependent on a myriad of factors, including peers and quality of life as well as perceived progress (36). Thus, to explain the trends in compliance in the two groups investigated in the present study, a qualitative study using one-to-one semi-structured interviews would be valuable.

An extended check-up interval between the two groups was also discussed before the start of the present study. A longer measuring interval of the sensor could have increased the storage capacity of the sensor and made an extended control interval possible. When the study was planned, only the 15 minutes interval for the sensors was known to the researchers. The option to change it to a longer interval of 20 minutes and increase the amount of data stored in the sensor was only presented to us after the planning had taken place and the study was set to start. In addition, a too-long check-up interval was considered unethical.

No significant differences were seen concerning overjet reduction and final overjet between the groups. In group 1 the overjet reduction was 5.2 mm and the final overjet 3.0 mm, and in group 2 the reduction was 4.7 mm, and the final overjet 3.8 mm. The molar relationship was normalized in the majority of the patients. The results are in line with other studies, which have shown that the Twin Block was effective, even if the patients used the appliance less than instructed (8,9,32). It might be beneficial to consider a shorter wear-time instruction than 12–14 hours per day to strengthen patient compliance. The patient’s perspective is one of the cornerstones in clinical research and an evaluation of the patient’s experience of different wear-time instructions would be interesting to investigate in the future.

Patients with an excessive overjet did not wear the appliance more compared to patients with a less excessive overjet. This result might be unexpected as large overjet could motivate the patient to comply, since a large overjet seems to be related to lower oral health-related quality of life and bullying (5,37). In addition, patients with excessive overjet did not experience trauma of the maxillary incisors more often compared to patients with less overjet. However, the small group sizes must be considered, and no instrument to evaluate the oral health-related quality of life of the patients was used in the present study.

Gender and age had no significant impact on wear-time in the present study. This is in accordance with earlier reports (19,38). However, boys showed, as in other studies, an increased risk of trauma of the maxillary incisors compared to girls (39).

The success rate in the total sample for treatments in the present study was 59 per cent, which is lower than presented in a recent study (6). A plausible explanation is that the optimal effects of functional appliances probably take place during peak mandibular growth, and the correlation between chronologic age and mandibular growth is poor (40). There is a great individual variation in the onset of the pubertal peak in mandibular growth, which affects the treatment outcome. In the present study, some patients may have passed the pubertal spurt and the peak in mandibular growth. This can explain the difference in success rate. It would perhaps have been more correct to correlate treatment outcome to other factors than chronological age, such as increase in skeletal maturity, body height, or dental development (38,41,42). The strict treatment protocol for successful treatment in the present study with a normalization of horizontal overjet (<4 mm) and a clear Class I molar relationship may have resulted in a lower success rate. If an improved molar relationship of half-cusp was accepted, the success rate increased to 74 per cent.

An analysis of the data of all patients included in the study disclosed a significant correlation between a successful treatment outcome and high wear-time between the third and the sixth months of treatment, regardless of a low wear-time at the beginning of the treatment. That is, patients with an initial low wear-time can improve in compliance over time and still end up with a successful treatment. To discontinue the treatment too soon could potentially cancel a successful treatment outcome.

### Strengths

The study was designed as an RCT that, according to the scientific hierarchy, delivers the most definitive answers regarding intervention effects by reducing the risk of confounding factors as these factors are evenly distributed between the two groups. The patients were followed prospectively for more than 6 months, which resulted in comprehensive data and great accuracy of the measurements.

The results were evaluated on an ITT basis and the attrition bias was relatively low since there were nine dropouts in total. In addition, the number of patients included in the study was still acceptable in relation to the power calculation. The present study used a validated method for measuring wear-time, which should reduce the risk of measurement error (22–24,26,32). The position of the TheraMon® sensor is reported to affect the accuracy of wear-time measurements. In the present study, the sensor was placed in a lingual position of the appliance to provide a more accurate measurement of the wear-time (23).

### Limitations and generalizability

It has been shown that single-centre trials tend to report higher treatment effects than multi-centre trials (42). Thus, the results of this study may contribute to a lower degree of generalizability.

The power analysis was made only on differences in overjet between the two groups and not on wear-time. The choice of overjet reduction as the primary outcome for power calculation was motivated by the intention to investigate a clinically-relevant outcome measure. Using wear-time as an outcome for the power calculation might be considered, as wear-time should correlate to the treatment outcome reduction in overjet.

To receive results as reliable as possible, it could be argued that the participants have to be unaware of how the compliance is measured. However, the ethical board considered it unethical not to inform the patients and custodians about how the wear-time was monitored. A systematic review showed that the Hawthorne effect could confound the results of a study because of the patient´s knowledge of being under surveillance (43). Nevertheless, it has been shown in previous studies that, even if the patients were aware of being monitored, they did not comply better (18,19). In addition, it has been shown that the Hawthorne effect decreases in trials conducted for more than 6 months (44). Hence, it seems probable that the patient’s knowledge of wear-time surveillance did not have a significant impact on the results of this study.

The Covid-19 pandemic arrived in Sweden in March 2020, and was therefore spreading in the country during the final months of the study (45). Some regions in Sweden locked down their dental care systems in the beginning of the pandemic, which affected the number of visits to the public and private dental clinics in a negative way. Children were less affected compared to adults and elderly. However, the dental services in Region Jönköping County, Sweden, remained operational during the pandemic. A comparison between 2019 and 2020 showed that in Region Jönköping regular check-ups in children aged 3–19 during week 2–51 was approximately 95 per cent of the check-ups made in 2019. Thus, the effect of the pandemic on orthodontic check-ups in this study was negligible (46).

Finally, the patients included in the trial were diverse in gender and age and represent a standard population of orthodontic patients. Therefore, the design and performance of this trial allows the results to be implemented in everyday orthodontic practice.

### Harm

No harm or adverse effects were recorded in the present study. Initial lateral open bites were registered during the treatment but were normalized by the end of treatment. The patients who were treated unsuccessfully were evaluated for later treatment with fixed appliances.

### Interpretation

A more frequent check-up interval of 4 weeks did not improve the patient´s wear-time or improve the reduction of overjet. The total number of visits increased by almost 25 per cent when the check-ups where performed every fourth week. Since there were no differences in the outcomes between the groups, a more frequent check-up interval seems to be unjustified and it is important to keep the number of visits as low as possible, to save time and costs for the patients and society. A well-designed multi-centre RCT on longer check-up intervals and patient compliance, treatment outcomes, and cost-effectiveness should be of interest to investigate in future studies.

## Conclusion

The present study showed that a more frequent check-up interval of 4 weeks instead of 6 weeks did not result in increased wear-time and/or improved treatment results in patients treated with the Twin Block appliance. A shorter wear-time than 12–14 hours per day seems to improve overjet, overbite, and molar relationship in patients who used the appliance.

## Data Availability

The data underlying this article cannot be shared publicly due to the privacy of individuals that participated in the study. The data will be shared on reasonable request to the corresponding author.
